# An essential role of Ffar2 (Gpr43) in dietary fibre-mediated promotion of healthy composition of gut microbiota and suppression of intestinal carcinogenesis

**DOI:** 10.1038/oncsis.2016.38

**Published:** 2016-06-27

**Authors:** S Sivaprakasam, A Gurav, A V Paschall, G L Coe, K Chaudhary, Y Cai, R Kolhe, P Martin, D Browning, L Huang, H Shi, H Sifuentes, M Vijay-Kumar, S A Thompson, D H Munn, A Mellor, T L McGaha, P Shiao, C W Cutler, K Liu, V Ganapathy, H Li, N Singh

**Affiliations:** 1 Department of Biochemistry and Molecular Biology, Georgia Regents University, Augusta, GA, USA; 2 Department of Cell Biology and Biochemistry, Texas Tech University Health Sciences, Lubbock, TX, USA; 3 Cancer Research Center, Georgia Regents University, Augusta, GA, USA; 4 Department of Pathology, Georgia Regents University, Augusta, GA, USA; 5 Department of Medicine, Georgia Regents University, Augusta, GA, USA; 6 Departments of Nutritional Sciences & Medicine, Pennsylvania State University, Medical Center, Hershey, PA, USA; 7 Department of Pediatrics, Georgia Regents University, Augusta, GA, USA; 8 Institute of Cellular Medicine, Newcastle University, Newcastle-upon-Tyne, UK; 9 Department of Immunology, University of Toronto, Toronto, ON, Canada; 10 College of Nursing, Georgia Regents University, Augusta, GA, USA; 11 Department of Periodontics, Georgia Regents University, Augusta, GA, USA

## Abstract

Composition of the gut microbiota has profound effects on intestinal carcinogenesis. Diet and host genetics play critical roles in shaping the composition of gut microbiota. Whether diet and host genes interact with each other to bring specific changes in gut microbiota that affect intestinal carcinogenesis is unknown. Ability of dietary fibre to specifically increase beneficial gut microbiota at the expense of pathogenic bacteria *in vivo* via unknown mechanism is an important process that suppresses intestinal inflammation and carcinogenesis. Free fatty acid receptor 2 (FFAR2 or GPR43) is a receptor for short-chain fatty acids (acetate, propionate and butyrate), metabolites of dietary fibre fermentation by gut microbiota. Here, we show *FFAR2* is down modulated in human colon cancers than matched adjacent healthy tissue. Consistent with this, *Ffar2*^*−/−*^ mice are hypersusceptible to development of intestinal carcinogenesis. Dietary fibre suppressed colon carcinogenesis in an Ffar2-dependent manner. Ffar2 played an essential role in dietary fibre-mediated promotion of beneficial gut microbiota, *Bifidobacterium* species (spp) and suppression of *Helicobacter hepaticus* and *Prevotellaceae*. Moreover, numbers of *Bifidobacterium* is reduced, whereas those of *Prevotellaceae* are increased in human colon cancers than matched adjacent normal tissue. Administration of *Bifidobacterium* mitigated intestinal inflammation and carcinogenesis in *Ffar2*^*−/−*^ mice. Taken together, these findings suggest that interplay between dietary fibre and Ffar2 play a key role in promoting healthy composition of gut microbiota that stimulates intestinal health.

## Introduction

Decreased intake of dietary fibre in modern era is associated with increased risk of colon cancers. Dietary fibre is fermented in the colon by gut microbiota into short-chain fatty acids (SCFAs; acetate, propionate and butyrate). SCFAs, specifically butyrate is well known for its anti-inflammatory and anti-carcinogenic effects in the gut.^1^ Butyrate enemas are known to suppress inflammation during ulcerative colitis, a risk factor for development of colorectal cancers.^1^ Moreover, recent studies demonstrate a reduction in butyrate-producing bacteria in feces of individuals with ulcerative colitis and colon cancers than healthy individuals.^2, 3^ This comparison may lead to inadequate information because there are several variables that are known to influence composition of gut microbiota that differ between healthy and colon cancer subjects such as genetic make up, lifestyle, age of individuals, dietary habits and therapeutic treatment. Therefore, an alternative method such as comparison between cancerous tissue and adjacent normal tissue may yield better information regarding role of different gut bacteria in promotion or suppression of colon cancers.

SCFAs interact with G-protein-coupled receptors 41 (GPR41 or FFAR1), FFAR2 and HCAR2 (also known as NIACR1 or GPR109A). FFAR1 and FFAR2 interact with all three SCFAs, whereas HACR2 interacts with butyrate only.^4, 5^ SCFAs are involved in homeostasis of regulatory T cells (Treg cells) in colon and modulation of intestinal carcinogenesis.^6, 7, 8, 9, 10, 11^ *Ffar2*^*−/−*^ mice reveal altered susceptibility to allergic airway inflammation in lung and colonic inflammation induced by dextran sulfate sodium (DSS), ethanol or trinitrobenzoic sulfonic acid.^12, 13, 14, 15^ FFAR2 expression in colon cancer cell lines promotes their apoptosis.^16^ However, the role of Ffar2 in regulation of intestinal carcinogenesis and underlying mechanism *in vivo* has not been investigated.

Fermentable dietary fibre (prebiotics) and *Bifidobacterium* spp (probiotics) improve gut epithelial barrier function, prevent apoptosis of intestinal epithelial cells, and suppress intestinal inflammation and carcinogenesis.^17, 18, 19, 20, 21, 22, 23^ *In vitro* studies using mixed cultures have shown that dietary fibre support growth of divergent groups of colonic bacteria such as *Clostridium*, *Bifidobacteria*, *Bacteroides*, *Enterococcus* and *Escherichia*.^24, 25^ In contrast, numerous human and animal studies show that dietary fibre consumption specifically and consistently promote only one group of gut bacteria, which is *Bifidobacterium* spp, and repress others such as *Enterococcus*, *Clostridium* and *Eubacterium*^24, 25^ suggesting that other factors regulate bifidogenic activity of dietary fibre *in vivo*.

It has been hypothesized that butyrate-producing gut bacteria play a critical role in dietary fibre-mediated suppression of colon carcinogenesis. However, studies show conflicting data about abundance of butyrate-producing bacteria in feces from colon cancers subjects than healthy individuals.^2, 26^ Similarly, both colon cancer-promoting and -suppressive effects of butyrate have been observed *in vivo* and this phenomenon has been termed as ‘butyrate paradox'.^10, 11^ *Fusobacterium nucleatum*, which is normally present in oral cavity of nearly all humans, has been shown to promote colon carcinogenesis.^27, 28, 29^ *F. nucleatum* is a butyrate producer.^30^ Butyrate-producing bacteria poorly utilize dietary fibre for their growth.^24, 31^ This may be the reason that a recent human study found that dietary fibre failed to significantly increase any single butyrate-producing bacterial species despite reducing the markers associated with colon carcinogenesis.^32^ On the other hand, *Bifidobacterium* spp are the best fermenters of dietary fibre.^24, 31^ Furthermore, *Bifidobacterium* ferment dietary fibre into acetate and lactate, which are utilized by butyrate-producing bacteria for growth and butyrate production and this process is termed as cross-feeding.^24, 31^ Therefore, if fermentation of dietary fibre is essential in suppression of colon carcinogenesis, being the primary fermenter of dietary fibre, *Bifidobacterium* must play an important role in it. In this report, we investigated whether Ffar2 regulates dietary fibre-mediated changes in gut microbiota and what is the impact of these changes on intestinal carcinogenesis. Our findings demonstrate the critical role of Ffar2 in maintaining a healthy composition of gut microbiota leading to suppression of intestinal carcinogenesis and suggest that Ffar2 signaling could be utilized as a potential target for therapeutic correction of gut microbiota to suppress intestinal carcinogenesis.

## Results

### Ffar2 is downregulated in human colon cancers

**Figure 1:**
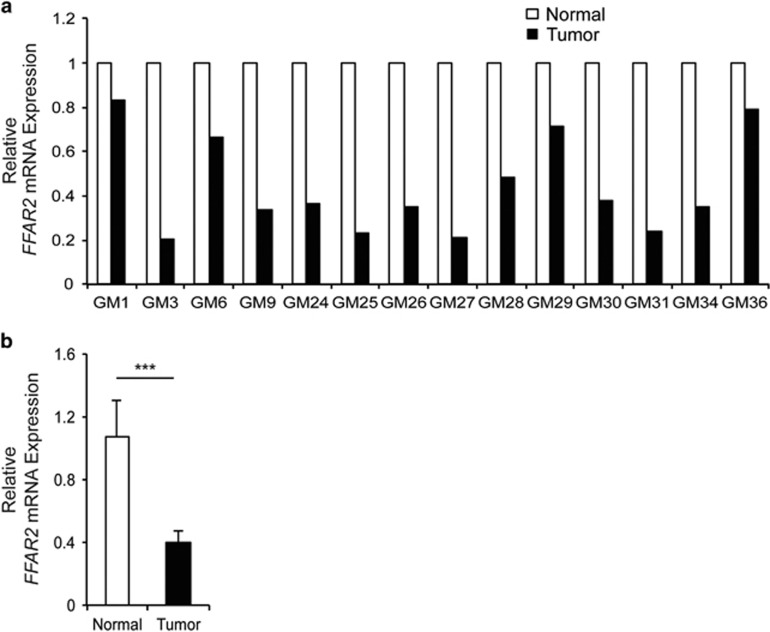
Expression of *FFAR2* is diminished in human colon cancers. (**a**) Expression of *FFAR2* was assessed by quantitative polymerase chain reaction in 14 colon cancer and matched adjacent normal tissue. (**b**) Data from **a** was pooled and re-plotted. ***P<0.0001.

To test the relevance of FFAR2 in colon cancers, *FFAR2* mRNA expression was analyzed in paired colon cancer and matched adjacent healthy tissue by quantitative polymerase chain reaction (qPCR). Figure 1a shows that expression of *FFAR2* mRNA was reduced by more than twofold in 71% (10 out of 14) of the colon cancers than adjacent normal tissue. When data was pooled for these samples, *FFAR2* expression was still significantly downregulated (by ~60%) in colon cancers than adjacent normal tissue (Figure 1b) (*P*<0.0001). Similarly, expression of *FFAR3* mRNA was also downregulated in colon cancer samples (Supplementary Figure 1). Same set of colon cancer samples as used in current study also exhibits decreased expression of HCAR2 (GPR109A).^33^

### Ffar2 suppresses inflammation-associated colon carcinogenesis

**Figure 2:**
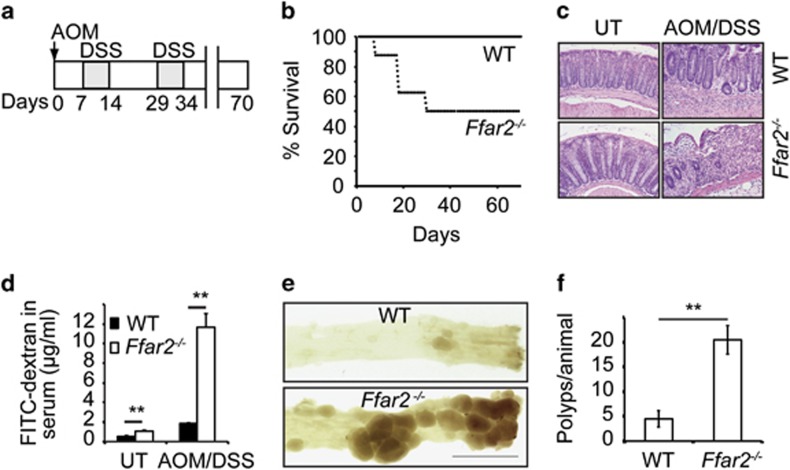
Ffar2 suppresses colon carcinogenesis. WT and *Ffar2*^*−/−*^ mice (littermates) were fed with conventional mouse chow. (**a**) Experimental model to induce inflammation-associated colon carcinogenesis. (**b**) Survival of WT and *Ffar2*^*−/−*^ mice subjected to AOM/DSS treatment as described in a (*n*=8 mice). (**c**) A representative photograph of H&E stained cross-section of colons of untreated (UT) or AOM/DSS treated WT and *Ffar2*^*−/−*^ mice. Original magnification 200 ×. (**d**) After the completion of first cycle of DSS, mice were gavaged with FITC-dextran and 6 h later FITC was quantified in serum (*n*=5 mice per genotype). (**e**) A representative photographs of luminal side of colons from WT and *Ffar2*^*−/−*^ animals on day 70 after AOM/DSS treatment. (**f**) Polyp burden in WT and *Ffar2*^*−/−*^ mice after AOM/DSS treatment (*n*=6 mice). A representative or pooled data from 2 experiments are shown. Error bars represent standard deviation. **P<0.002.

Since Ffar2 was downregulated in colon cancers, an inflammation-associated colon carcinogenesis model was used to test the role of Ffar2 in colonic inflammation and carcinogenesis. *Ffar2*^*−/−*^ mice and their wild-type (WT) littermates were injected with the colon-specific carcinogen azoxymethane (AOM) intraperitoneally (i.p.) followed by two cycles of DSS in drinking water as shown in Figure 2a. All the WT littermates treated with AOM and DSS (AOM/DSS) survived until the experimental endpoint. However, 50% of the *Ffar2*^*−/−*^ mice died within 30 days of treatment (Figure 2b). In contrast to WT littermates, *Ffar2*^*−/−*^ mice exhibited drastic reduction in body weight, and severe diarrhea during the first cycle of DSS (Supplementary Figures 2a and b). Similarly, higher weight loss was also observed in *Ffar2*^*−/−*^ mice than WT counterparts during second cycle of DSS treatment (Supplementary Figure 2a). Following AOM/DSS treatment, colons of *Ffar2*^*−/−*^ mice exhibited drastically increased loss of epithelium, crypt structure and heightened infiltration of colonic mucosa by immune cells relative to WT littermates resulting in higher histopathological scores in the former group (Figure 2c and Supplementary Figure 2c). Following oral gavage of fluorescein isothiocyanate (FITC)-dextran to AOM/DSS treated animals, sixfold more FITC-dextran was translocated to systemic circulation of *Ffar2*^*−/−*^ mice than WT littermates, revealing increased gut epithelial barrier dysfunction in former group (Figure 2d). Consistent with these findings, staining with claudin-3, a tight junction protein revealed denudation of epithelium following AOM/DSS treatment in *Ffar2*^*−/−*^ mice (Supplementary Figure 2d). In addition, colons of AOM/DSS treated *Ffar2*^*−/−*^ mice exhibited significantly higher weight per centimeter length than WT littermates, presumably due to thickening of colon in *Ffar2*^*−/−*^ mice (Supplementary Figure 2e). Expression of several pro-inflammatory molecules such as IL-1β, IL-17a, Ccl-2, Cox-2, Cxcl-1, Cxcl-2 and IL-12 that promote colonic inflammation was significantly increased in colons of *Ffar2*^*−/−*^ mice than WT mice after AOM/DSS treatment (Supplementary Figure 3). At the experimental endpoint, colons of mice were harvested, opened longitudinally and analyzed for number of polyps. Colons of AOM/DSS treated *Ffar2*^*−/−*^ mice had ~4.5 time more number of polyps than WT littermates mice (Figures 2e–f). In addition, polyps in colons of *Ffar2*^*−/−*^ mice were larger in size than WT littermates (Supplementary Figure 2f). Collectively, these data demonstrate that Ffar2 is a key receptor that suppresses inflammation-associated colon carcinogenesis.

### Ffar2 controls intestinal carcinogenesis in *Apc*^*Min/+*^ mice

**Figure 3:**
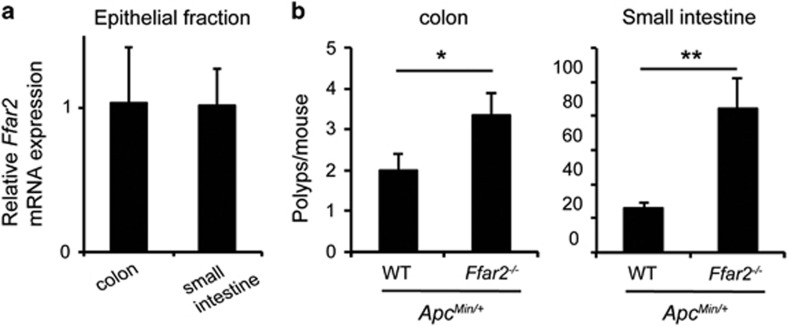
Ffar2 regulates tumorigenesis in *Apc*^Min/+^ mice. (**a**) *Ffar2* mRNA expression by colonic and small intestinal epithelium (*n*=3 mice). A representative of two experiments is shown. (**b**) Polyp burden in colon and small intestine of *Apc*^Min/+^ (*n*=7) and *Ffar2*^*−/−*^ *Apc*^Min/+^ mice (*n*=6). *P<0.01, **P<0.002.

Next, we analyzed the role of Ffar2 in a genetic model of intestinal carcinogenesis. Approximately 85% of the sporadic human colon cancers harbor mutations in the adenomatous polyposis coli (*APC*) gene.^34^ Heterozygous mutation in *Apc* that is present in *Apc*^*Min/+*^ mice results in expression of a truncated protein. These mice develop of numerous polyps in small intestine and colon. We found that Ffar2 is expressed in epithelial cells throughout the small intestine (Figure 3a). *Ffar2*^*−/−*^*Apc*^*Min/+*^ mice developed significantly more polyps in colon than *Apc*^*Min/+*^ mice (Figure 3b). SCFAs are generated in the cecum and colon, and therefore it has been hypothesized that they will inhibit carcinogenesis only in the cecum and colon. However, the concentrations of SCFAs in ileum is ~10 mM.^35^ Ec50 of SCFA to induce Ca^++^ flux or GDP to GTP exchange via Ffar2 is <1 mM,^4^ therefore it is highly possible that at steady state there is spontaneous signaling via Ffar2 in small intestine. At 5 month of age, *Ffar2*^*−/−*^*Apc*^*Min/+*^ mice developed significantly more polyps in small intestine than age matched *Apc*^*Min/+*^ mice (Figure 3b). These data demonstrate that Ffar2 signaling regulates development of intestinal carcinogenesis induced by germ line mutation of *Apc.*

### Decreased numbers of *Bifidobacterium* spp and increased numbers of *Prevotellaceae* and *H. hepaticus* in gut microbiota of *Ffar2*^*−/−*^ mice

**Figure 4:**
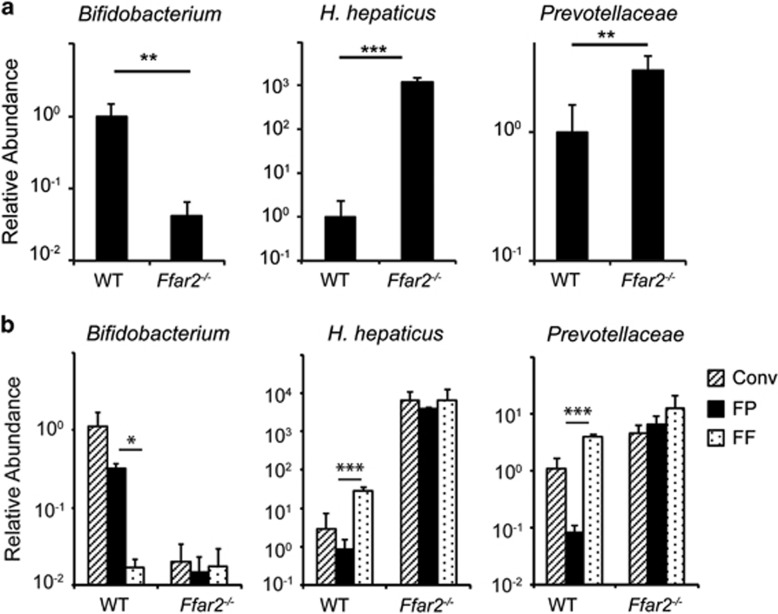
An essential role of Ffar2 in dietary fibre-mediated promotion of *Bifidobacterium* and suppression of *Prevotellaceae* and *H. hepaticus*. (**a**) Fecal DNA from *Ffar2*^*−/−*^ mice and their WT littermates were evaluated for abundance of indicated gut microbiota. (**b**) *Ffar2*^*−/−*^ mice and their WT littermates maintained on conventional diet (Conv) were fed with FF or FP diets. The abundance of indicated bacterial groups in feces was quantified 1 day before (Conv) switching the diet or after 1 week on experimental diets (FF or FP). Shown is the relative abundance of indicated bacterial groups to that of total bacteria. *P<0.05, **P<0.01, ***P<0.0001. (*n*=4 mice). A representative of two experiments is shown.

Numerous studies have documented that gut microbiota dysbiosis enhances risk for development of intestinal inflammation and cancers.^2, 3, 19, 36^ We hypothesized that gut microbiota dysbiosis enhances risk of colon carcinogenesis in *Ffar2*^*−/−*^ mice. To test our hypothesis, we analyzed the presence of different bacterial groups among gut microbiota in *Ffar2*^*−/−*^ mice and their WT littermates. Feces of WT littermates contained ~24-fold more *Bifidobacterium* spp among gut microbiota than *Ffar2*^*−/−*^ mice (Figure 4a). Compared with feces of WT littermates, those of *Ffar2*^*−/−*^ mice exhibited ~1200-fold more *H hepaticus* (Figure 4a). Similarly, members belonging to the *Prevotellaceae* family (Phylum, Bacteroidetes) were present in significantly higher numbers in feces of *Ffar2*^*−/−*^ mice than WT littermates (Figure 4a). Bacterial members from phyla Firmicutes and Bacteroidetes dominate gut microbiota.^37^ Firmicutes in gut microbiota are mainly represented by members belonging to *Lactobacillus* spp, *Clostridium leptum*, segmented filamentous bacteria and *Eubacterium rectales* groups.^38^ Feces from WT littermates and *Ffar2*^*−/−*^ mice contained similar frequencies of bacteria from these groups (Supplementary Figure 4a). Similarly, *Bacteroides* and mouse intestinal *Bacteroides* groups, both from phylum Bacteroidetes^38^ were present at comparable levels in feces of WT and *Ffar2*^*−/−*^ littermates (Supplementary Figure 4a). In accordance with fecal microbiota, number of *Bifidobacterium* was decreased, whereas those of *H hepaticus* and *Prevotellaceae* were increased among microbiota attached to colons of *Ffar2*^*−/−*^ mice than WT littermates (Supplementary Figure 4b). Comparable numbers of bacteria belonging to *Bacteroides*, mouse intestinal *Bacteroides*, *C leptum*, *E rectales and Lactobacillus* groups were attached to the colons of WT and *Ffar2*^*−/−*^ littermates (Supplementary Figure 4b). Taken together, these data demonstrate that the composition of gut microbiota from *Ffar2*^*−/−*^ mice is enriched in favor of bacteria that promote intestinal inflammation and cancers, whereas the numbers of bacteria that suppress intestinal inflammation and cancers are reduced.

### Essential role of Ffar2 in dietary fibre-mediated promotion of *Bifidobacterium* spp and decrease of *Prevotellaceae* and *H hepaticus* among gut microbiota

Human as well as animal studies have shown that dietary fibre consumption specifically and consistently increases number of only *Bifidobacterium* spp in gut.^24^ We hypothesized that Ffar2 plays an obligatory role in dietary fibre-mediated promotion of *Bifidobacterium* spp. To test this hypothesis, we fed *Ffar2*^*−/−*^ mice and their WT littermates with a fibre free (FF) or fibre plus (FP) diets. FP diet contained 2% inulin, 2% pectin and 1% cellulose as source of dietary fibre, whereas FF diet completely lacks dietary fibre, otherwise all other components between these two diets are exactly same. The composition of gut microbiota in feces of *Ffar2*^*−/−*^ mice and their WT littermates were evaluated 1 week later. Fecal microbiota changed between conventional diet and FF or FP diet (Figure 4b). Conventional diet contains many components such as soyabean meal, ground corn, flaked corn, molasses, wheat middling, ground wheat etc. Compositions of these components are not exactly defined. On the other hand, FF and FP diets are made from purified and defined components. Thus, conventional diet differs from FF or FP with respect to several food components. Therefore, it is difficult to assign any changes in gut microbiota from conventional diet to FF or FP diet to a particular food component. Feces of FF diet-fed WT animals contained ~17-fold less number of *Bifidobacterium spp* than FP diet-fed WT littermates (Figure 4b). In sharp contrast, feces of *Ffar2*^*−/−*^ mice fed with either FF or FP diet contained similar numbers of *Bifidobacterium spp*, which were significantly lower than numbers present in feces of FP-fed WT littermates (Figure 4b). *Prevotellaceae* and *H hepaticus* were present in similar number in feces of FF-fed WT, FF- or FP-fed *Ffar2*^*−/−*^ mice, which was significantly higher than that present in feces of FP-fed WT littermates (Figure 4b). In contrast, dietary fibre increased *Bacteroides* in gut microbiota in an Ffar2-independent manner (Supplementary Figure 5). Numbers of mouse intestinal *Bacteroides* and *Lactobacillus* spp among gut microbiota were unaffected by dietary fibre content (Supplementary Figure 5). Consistent with previous studies,^39, 40^ abundance of *E. rectales* and *C. leptum* group was modestly decreased by dietary fibre in WT mice (Supplementary Figure 5). Collectively, these data demonstrate that Ffar2 is indispensable for dietary fibre-mediated promotion of *Bifidobacterium* spp and suppression of *H hepaticus and Prevotellaceae* among gut microbiota.

### An essential role of Ffar2 in dietary fibre-mediated suppression of colonic inflammation and carcinogenesis

**Figure 5:**
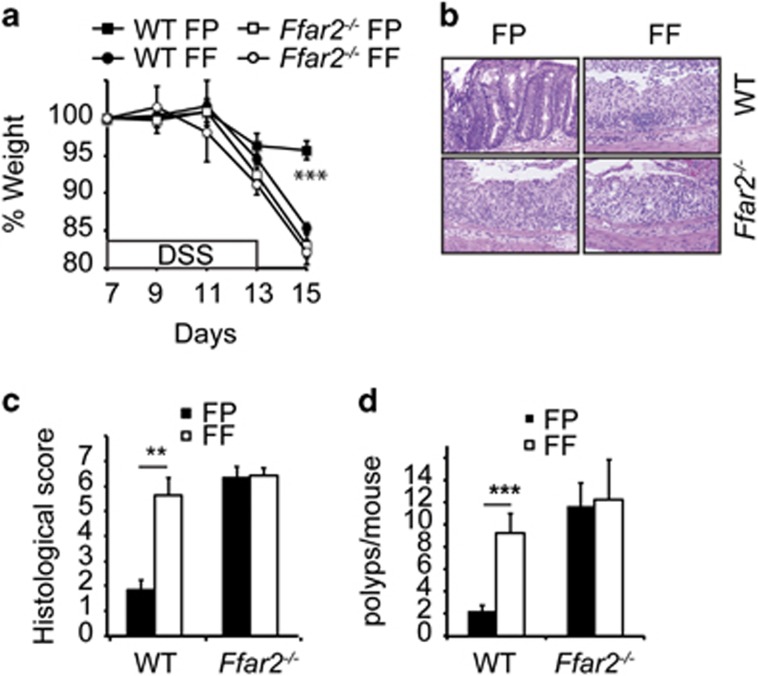
An essential role of Ffar2 in dietary fibre-mediated suppression of colonic inflammation and carcinogenesis. WT and *Ffar2*^*−/−*^ mice (littermates) were fed with FF and FP diets and 1 month later were injected with AOM (i.p.). After 1 week, all the mice were given 1.5% DSS in drinking water for next 6 days. (**a**) Weight loss during and after DSS treatment. (**b**) A representative photograph of H&E stained cross-section of colons of mice after first cycle of DSS as treated in **a**. (**c**) Histopathological score (inflammation+epithelial damage) of colons from mice 4 days after completion of DSS treatment. (**d**) Polyp burden in colons of indicated mice at the end of experiment. A representative of two experiments is shown (*n*=4 mice). **P<0.01, ***P<0.0001.

Next, role of Ffar2 in dietary-mediated suppression of colon carcinogenesis in AOM/DSS model was tested. FF diet-fed WT littermates exhibited dramatically more weight loss and diarrhea than FP diet-fed counterparts, demonstrating an essential role for dietary fibre in AOM/DSS-mediated disease (Figure 5a and Supplementary Figure 6a). Colons of WT mice fed with FF diet showed severe pathology with erosion of epithelial layer, ulceration, loss of crypt structures and higher histopathological scores than FP diet-fed counterparts (Figures 5b and c). Consistent with this, depletion of dietary fibre led to the development of significantly higher numbers of colonic polyps in AOM/DSS-treated WT littermates (Figure 5d). The ability of dietary fibre to suppress AOM/DSS-induced weight loss, diarrhea and development of colonic polyps was dependent on Ffar2, because *Ffar2*^*−/−*^ mice fed with either FF or FP diets exhibited weight loss, diarrhea and colonic polyps, which were comparable with outcomes observed in WT littermates fed with FF diet (Figures 5a–d). Taken together these data demonstrates a critical role of Ffar2 in dietary fibre-mediated suppression of colonic inflammation and carcinogenesis.

### *Bifidobacterium* suppresses colonic inflammation and carcinogenesis in *Ffar2*^*−/−*^ mice

**Figure 6:**
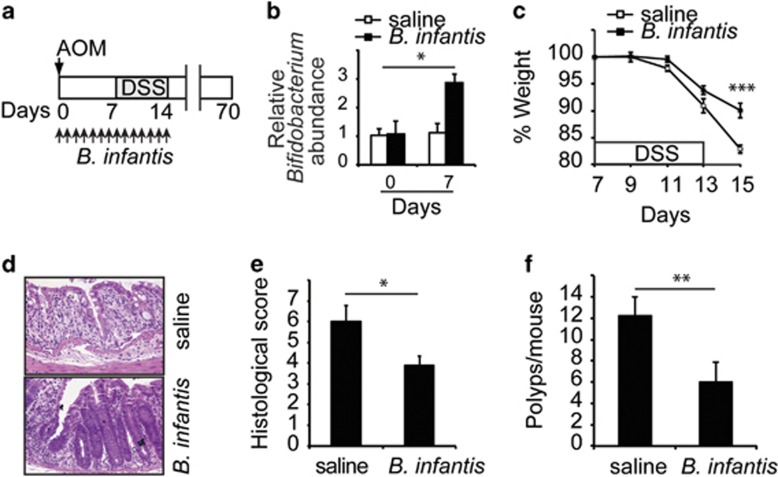
Bifidobactreia suppress colonic inflammation and carcinogenesis in *Ffar2*^*−/−*^ mice. (**a**) *Ffar2*^*−/−*^ mice fed with FP diet and 1 month later were challenged with AOM/DSS and *B. infantis* as shown. *B. infantis* was gavaged everyday (2 × 10^8^ cfu/mouse) as indicated from day of AOM injection till the completion of DSS treatment. (**b**) Relative abundance of *Bifidobacterium* in mice gavaged with *B. infantis* or saline as in **a**. Shown is the weight loss (**c**) during and after DSS challenge. (**d**) A representative photograph of H&E stained cross-section of colons of mice 4 days after cessation of DSS treatment. (**e**) Histopathological score (inflammation+epithelial damage) of colons. (**f**) Polyp burden in colons of mice treated as described in a at experimental endpoint. A representative of two experiments is shown (*n*=4 mice). *P<0.05, **P<0.01, ***P<0.0001.

A recent study demonstrates that co-housing of *Ffar2*^*−/−*^ mice with WT mice attenuate DSS-induced colonic inflammation in *Ffar2*^*−/−*^ mice.^41^ This finding suggested that (1) an unknown beneficial gut bacteria that got transferred from WT to *Ffar2*^*−/−*^ mice during co-housing protected *Ffar2*^*−/−*^ mice from colonic inflammation and (2) *Ffar2*^*−/−*^ mice are deficient in this gut bacteria. However, identity of this beneficial gut bacterium is unknown. Data presented above argues in favor of *Bifidobacterium* spp being these beneficial gut bacteria. Therefore, the ability of *Bifidobacteria* to suppress colonic inflammation and carcinogenesis in *Ffar2*^*−/−*^ mice was tested. FP-fed *Ffar2*^*−/−*^ mice were gavaged orally with *Bifidobacterium longum subsp. infantis* (ATCC 15697, *B infantis*) and challenged with AOM/DSS as described in Figure 6a. Number of *Bifidobacterium* in feces significantly increased in mice gavaged with *B. infantis* than saline (Figure 6b). *B infantis* significantly suppressed weight loss, diarrhea, inflammation and development of polyps in colon of *Ffar2*^*−/−*^ mice (Figures 6c–f and Supplementary Figure 6b). Collectively, these data demonstrate that *Bifidobacterium* deficiency enhances risk for development of colonic inflammation and carcinogenesis in *Ffar2*^*−/−*^ mice.

### Decreased numbers of *Bifidobacterium* and increased numbers of *Prevotellaceae* in human colon cancers

**Figure 7:**
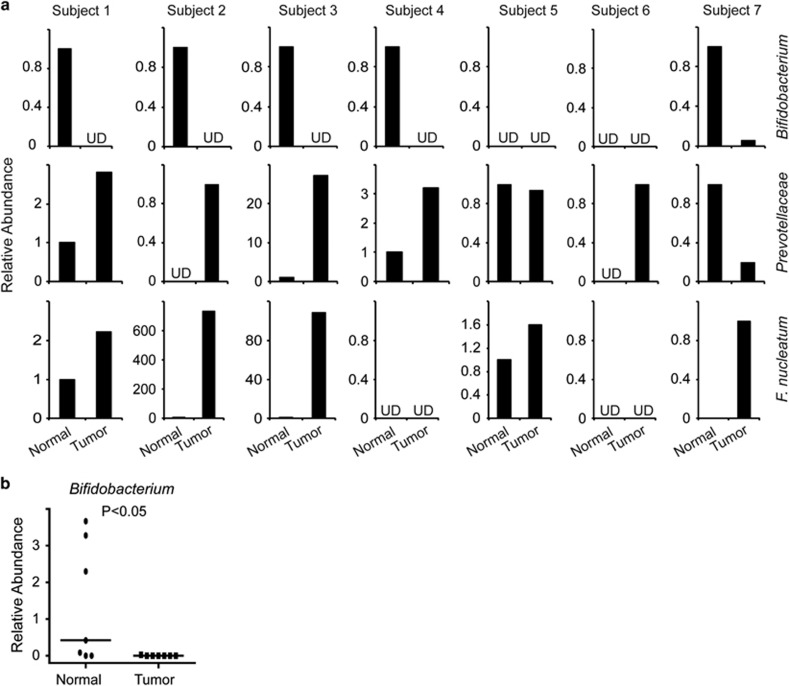
Decreased abundance of *Bifidobacterium spp* in human colon cancers. (**a**) Relative abundance of indicated bacterial groups in DNA extracted from colon cancers and matched adjacent normal tissue was measured by quantitative polymerase chain reaction. (**b**) Relative levels of *Bifidobacterium* in colon cancer and matched adjacent normal tissue. The horizontal line represents the median value. *P<0.05. UD, undetectable. Statistical significance was calculated using Mann–Whitney test with two-tailed analysis.

Based on our findings above, we evaluated the presence of *Bifidobacterium* and *Prevotellaceae* in human colon cancers and adjacent normal tissue. *Bifidobacterium* spp were readily detectable in most (5 out of 7) of the normal tissue, whereas it was either undetectable or present at drastically reduced numbers in cancerous tissue than corresponding adjacent normal tissue (Figure 7a). When all the seven paired samples were analyzed together, significantly higher number of *Bifidobacterium* was present in matched adjacent normal tissue than tumor (Figure 7b). In contrast, *Prevotellaceae* was present in higher numbers in most of the cancerous tissue than matched adjacent normal tissue (Figure 7). Consistent with previous studies,^27, 28, 29^ *F nucleatum* was present in higher numbers in colon cancer tissues than corresponding adjacent normal tissue (Figure 7a). Analysis of feces from healthy and colon cancer subjects have shown a decrease of butyrate-producing bacteria in later. However, *F nucleatum* is a butyrate producer and its numbers are increased in colon cancers than normal tissue.^27, 28, 29, 30^ Therefore, we analyzed the presence of other butyrate-producing groups, such as *C leptum* and *C coccoides/E rectale* in colon cancers and matched adjacent normal tissue. Surprisingly, we found that numbers of *C coccoides/E rectale* group was drastically increased in four out of seven colon cancers than adjacent normal tissue (Supplementary Figure 7). However, we could not find a clear correlation for abundance of *C leptum* or *F prausnitzii* between cancerous and matched normal adjacent tissue (Supplementary Figure 7). Collectively these data demonstrate that the number of *Bifidobacterium* is decreased, whereas that of *Prevotellaceae* is increased in colon cancers than matched normal tissue.

## Discussion

In the current study, we identified decreased numbers of *Bifidobacterium* spp and increased numbers of *Prevotellaceae* in colon cancers than matched adjacent normal tissue. One of the most important findings of this study is that Ffar2 is indispensable for dietary fibre-mediated promotion of *Bifidobacterium* spp and inhibition of *H hepaticus* and *Prevotellaceae* among gut microbiota. Increase in *Bifidobacterium* spp in gut is not specific to the mixture of inulin, pectin and cellulose as source of dietary fibre used in our study, because different types of dietary fibre such as lactulose, trans-galactooligosaccharides and fructooligosaccharides increase *Bifidobacterium* spp in human gut.^24, 25^ The effect of dietary fibre in enhancing numbers of *Bifidobacterium* spp varies among individuals.^25^ Based on our findings, it will be important to investigate whether altered expression or polymorphism of Ffar2 relates to dietary fibre-mediated promotion of *Bifidobacterium* spp in humans. Although host genetics and diet can independently affect gut microbiota,^42, 43^ our data demonstrate that interaction between both diet and host genes also play a critical role in influencing the composition of gut microbiota.

In contrast to comparisons between feces of healthy and colon cancer subjects, our data comparing colon cancer and adjacent normal tissue found an increase in butyrate-producing bacteria belonging to *E rectale* group in most of the colon cancers, which is in line with a previous study.^44^ *F nucleatum*, which is associated with colon cancer tissue and promotes colon carcinogenesis, is a butyrate producer. Evidences have been presented for both tumor-promoting and -suppressing effect of butyrate in colon.^10, 11^ Tumor-suppressing activity of butyrate has been largely attributed to its ability to inhibit HDAC activity. At single cell level, butyrate can induce apoptosis in colon cancer cells; however, at tissue or organ level, continuous exposure to butyrate will lead to toxicity towards other cells such as underlying connective tissue in colon, which may induce inflammation and/or promote metastasis. In fact, it has been proposed that *F nucleatum* produced butyrate plays a key role in induction of plaques and pathologies in dental tissues by inhibiting growth of fibroblast.^30^ Therefore, a lot of care should be taken in to considerations, such as effects of butyrate at tissue or organ level, effects of other cellular, molecular, metabolic or virulence factors of butyrate-producing bacteria in evaluating their role in colon carcinogenesis.

In summary, data presented in this study demonstrate that Ffar2 signaling promotes intestinal health by stimulating growth of beneficial bacteria such as *Bifidobacterium* and by decreasing the number of harmful gut microbiota such as *H hepaticus* and *Prevotellaceae*. It is evident that SCFA receptors Ffar2 and Hcar2 induce non-redundant mechanisms such as inflammasome activation, homeostasis of colonic Treg cells and promoting a healthy composition of gut microbiota,^8, 9, 10, 41^ which could explain how a defect in even one of the these receptors may result in reduced efficacy of dietary fibre to suppress intestinal carcinogenesis. A better understanding of all the signaling pathways induced by SCFA and dietary fibre will be useful in designing precise and better therapeutic strategies to prevent and/or treat intestinal inflammation and cancers.

## Materials and methods

### Mice and diets

C57BL/6J mice were obtained from Jackson laboratory. *Ffar2*^*+/−*^ mice on C57BL/6 background were obtained from Deltagen. *Ffar2*^*+/−*^ mice were bred to obtain *Ffar2*^*−/−*^ mice. Both males and females of ages between 3 and 6 months were used in study. Animals were maintained on conventional rodent chow, TD-8604 (Harlan Laboratories, Madison, WI, USA) unless stated. FF (TD-00278) and FP (TD-130715) mouse chows were purchased from Harlan Laboratories. Formulation for FF diet is as follows: Lactalbumin 20.5%, DL-Methionine 0.22%, Dextrose monohydrate 52.839%, Maltodextrin 15%, Soybean Oil 5%, Mineral Mix AIN-93G 3.5%, Vitamin Mix AIN-93 1.5%, Potassium phosphate monobasic 0.84%, Calcium carbonate 0.3%, Choline Bitartrate 0.3% and TBHQ antioxidant 0.001%. FP diet is similar to FF diet, with 5% dietary fibre (1% cellulose, 2% pectin and 2% inulin) added. *Bifidobacterium longum* subsp. *Infantis* ATCC 15697 (*B infantis*) was purchased from American Type Culture Collection (ATCC). Where indicated, mice were gavaged orally with 2 × 10^8^ CFU of *B infantis* in 100 μl of sterile saline buffered with 5% sodium bicarbonate. The Institutional Animal Care and Use Committee, Georgia Regents University approved all animal procedures.

### Colitis-associated colon cancer model

Mice were injected intraperitoneally (i.p.) with AOM, (Sigma, St Louis, MO, USA) at dose of 10 mg/kg body weight in phosphate buffered saline. After 7 days, mice were fed 2% DSS, (MP Biochemicals, Santa Ana, CA, USA) molecular mass 36 000–50 000 Da in the drinking water for 7 days, followed by 15 days of regular water. This cycle was repeated twice. In experiments involving FP or FF diet, mice received a single cycle of 1.5% DSS in drinking water. Mice were monitored for weight changes, diarrhea and rectal bleeding. Diarrhea was scored as (0) normal stool, (1) soft but formed pellet, (2) very soft pellet, (3) diarrhea (no pellet) or (4) dysenteric diarrhea. Rectal bleeding was recorded as (0) no bleeding, (2) presence of occult blood in stool or (4) gross macroscopic bleeding. Seventy days following AOM injection, colonic lumens of mice were evaluated for polyp number and size.

### Measurement of gut epithelial barrier function

Gut epithelial barrier function was evaluated using FITC-dextran, 4kD (Sigma). Mice were administered an oral gavage of FITC-dextran as a permeability tracer at a dose of 50 mg/100 g of body weight. Six hours later, mice were bled, serum was collected and FITC-dextran in serum was measured by fluorescence spectrophotometry (SpectraMax, Molecular Devices, Sunnyvale, CA, USA). FITC-dextran concentration was determined from standard curves generated by serial dilution of known concentrations of FITC-dextran in control serum ran in parallel.

### Immunohistochemistry

Colons were excised and cleaned with phosphate buffered saline followed by fixation in neutral buffered formalin (Thermo Fisher, Waltham, MA, USA). The fixed colon tissues were embedded in paraffin and 5 μm thick sections were sliced and placed on glass microscope slides. Immunohistochemical staining was performed as described previously.^8^ Briefly, sections were deparaffinized, followed by antigen retrieval using the antigen retrieval solution (Agilent Technologies, Santa Clara, CA, USA). Endogenous peroxidase activity was quenched with 3% H_2_O_2_ in phosphate buffered saline for 10 min. Sections were stained using specific primary antibodies and Vectastain ABC kit and diaminobenzidine (Vector Laboratories, Burlingame, CA, USA). Counterstaining was performed with hematoxylin and stained sections were visualized by Leica DM550B microscope. Alternatively, in some experiments, fluorescent dye-labeled secondary antibody was used and sections were visualized by LSM 510 (Zeiss) confocal microscopy.

### Isolation of RNA, DNA and quantitative PCR

Total RNA was extracted from colon tissue using TRIzol reagent (Thermo Fisher). The high capacity cDNA synthesis kit (Thermo Fisher) was used for synthesis of cDNA. Relative amounts of mRNAs were measured using polymerase chain reaction (PCR) and StepOne Real-time PCR system (Thermo Fisher) on cDNA samples. Gene expression was quantified using ΔΔCT method.

### Colon cancer and matched adjacent normal tissue

Anonymous human colon cancer tissue and matched adjacent normal tissue from all demographics were obtained after informed consent and approval from the Institutional Review Board. The samples were collected over a period of time at Georgia Regents Medical Center Augusta, GA, USA. PCR primer for detection of *FFAR2* has been described.^45^

### Quantification of gut microbiota

Feces (~100 mg) were suspended with 710 μl of disruption buffer (200 mM NaCl, 200 mM Tris pH 8.0, 20 mM EDTA pH 8.0 and 6% SDS) and 500 μl of phenol/chloroform/isoamyl alcohol, pH 8.0 inside tubes containing Zirconium beads (0.1 mm diameter, Benchmark Scientific, Edison, NJ, USA). The mixture was homogenized using a Beadbeater (BioSpec, Bartlesville, OK, USA) for 2 cycles of 2 min each. Sample was centrifuged at 7000 g for 3 min and aqueous phase was collected and a second round of phenol/chloroform/isoamyl extraction was performed. DNA from the clear aqueous phase was precipitated using sodium acetate and isopropanol. Dried DNA pellet was dissolved in TE buffer. Quantitative polymerase chain reaction using group-specific PCR primers were used to quantify relative abundance of different groups to the total gut microbiota as reported previously^38, 46, 47^ and the sequences of PCR primers are provided in Supplementary Table 1.

### Statistical analysis

Statistical significance was calculated using *T*-test with two-tailed analysis unless stated otherwise. No method was used to predetermine the sample size. Experiments were performed and analyzed in non-randomized and non-blinded fashion.
