# Supplementary material for: An essential role of Ffar2 (Gpr43) in dietary fibre-mediated promotion of healthy composition of gut microbiota and suppression of intestinal carcinogenesis
Source: Oncogenesis. 2016 Jun 27;5(6):e238–. doi: 10.1038/oncsis.2016.38 (PMC4945739; doi:10.1038/oncsis.2016.38)
Supplement: Supplementary Information [file oncsis201638x1.doc]

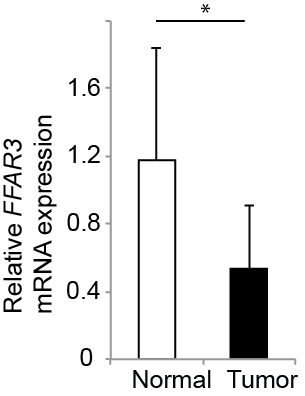


**Supplementary Figure 1.** Expression of *FFAR3* mRNA in human colon cancer (tumor) and matched adjacent normal (normal) tissue from samples shown in Figure 1. N=14.

* P<0.05.


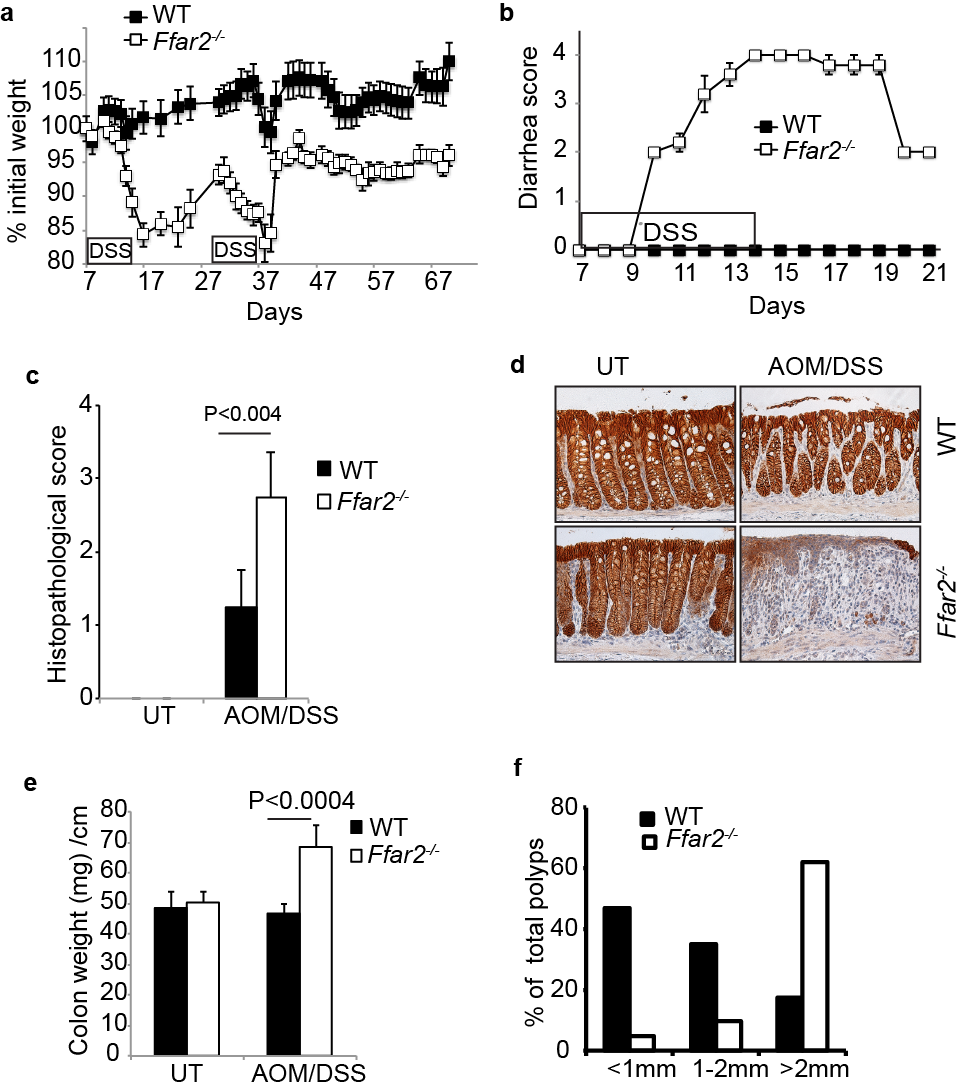


**Supplementary Figure 2.** Ffar2 attenuates AOM/DSS induced colonic inflammation and carcinogenesis. Weight change (a) and diarrhea (b) in AOM/DSS treated WT and *Ffar2^-/-^* mice during and after DSS regime (n=4). (c) Histopathological score (inflammation + epithelial damage) (n=5). (d) Claudin-3 staining of colonic sections from untreated (UT) or AOM/DSS-treated mice of indicated genotype (original magnification, 200X) (e) Weight of colons per centimeter length from WT and *Ffar2^-/-^* mice treated as indicated (n=4). (f) Size distribution of polyps developed in colons of AOM/DSS treated WT and *Ffar2^-/-^* mice.


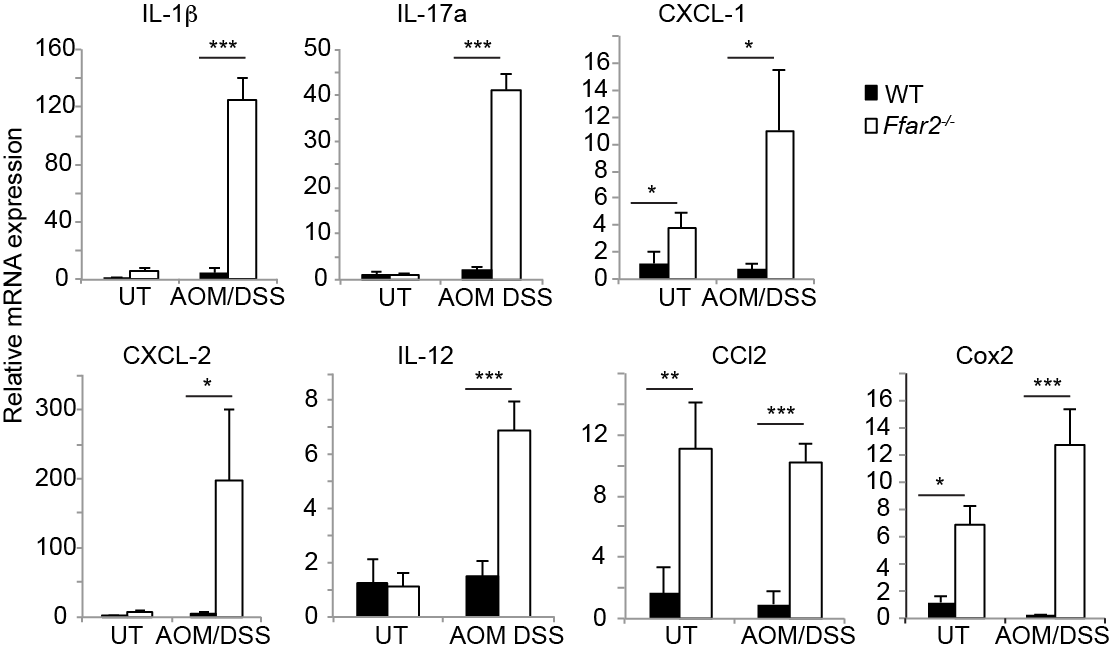


**Supplementary Figure 3.** Expression of inflammatory genes in untreated and AOM/DSS treated and untreated WT and *Ffar2^-/-^* mice. Value are from 3 independent experiments. *P<0.05, ** P<0.01, *** P<0.0001


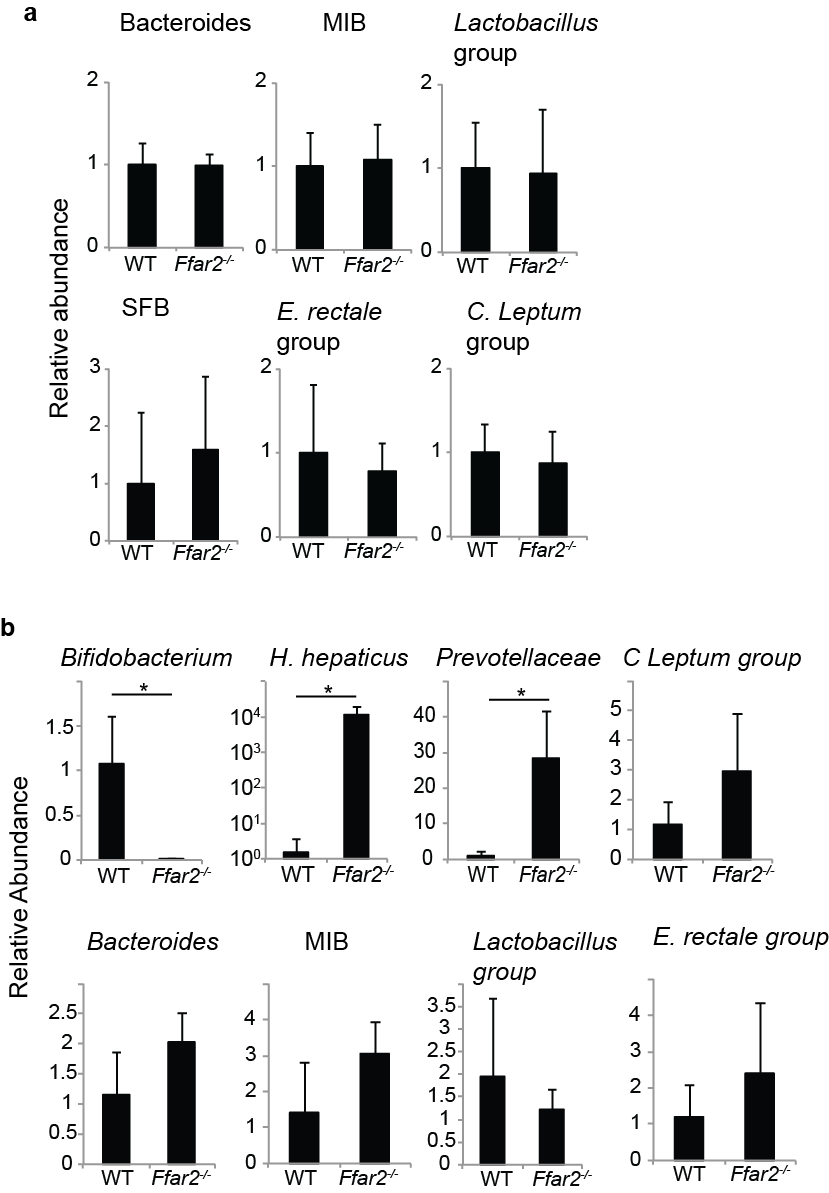


**Supplementary Figure 4.** Relative presence of different bacterial groups to that of total bacteria in feces (a) and attached to colon (b) of WT and *Ffar2^-/-^* mice fed with conventional mouse chow (n=4). * P<0.05. A representative of two experiments is shown.


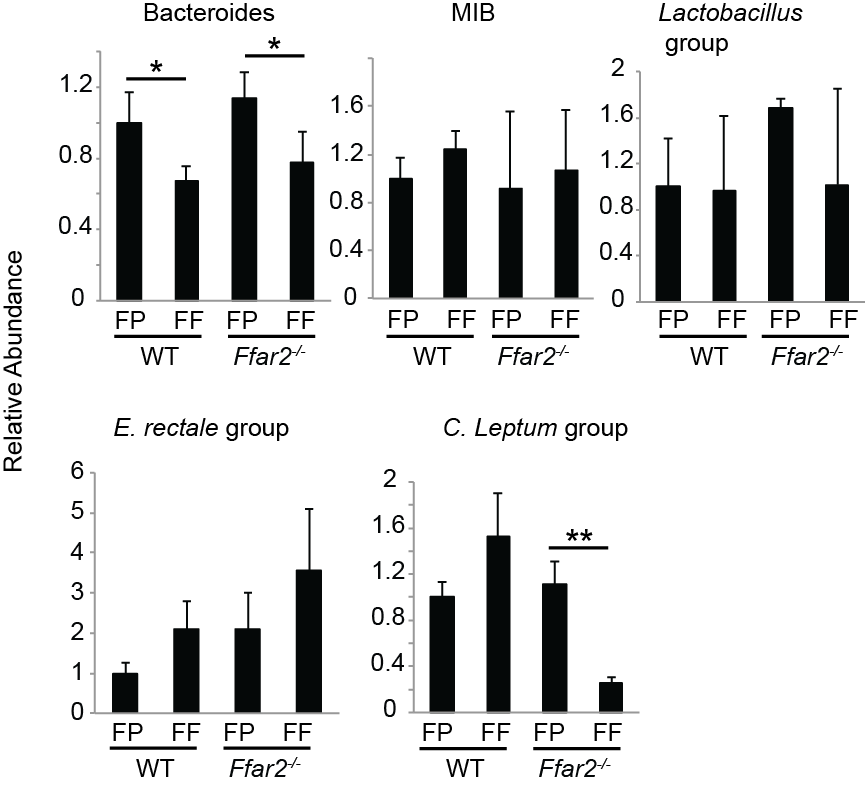


**Supplementary Figure 5.** Presence of indicated gut-bacterial groups to that of total bacteria in feces of WT and *Ffar2^-/-^* mice fed with FF or FP diets (n=4). *P<0.05, ** P<0.01.


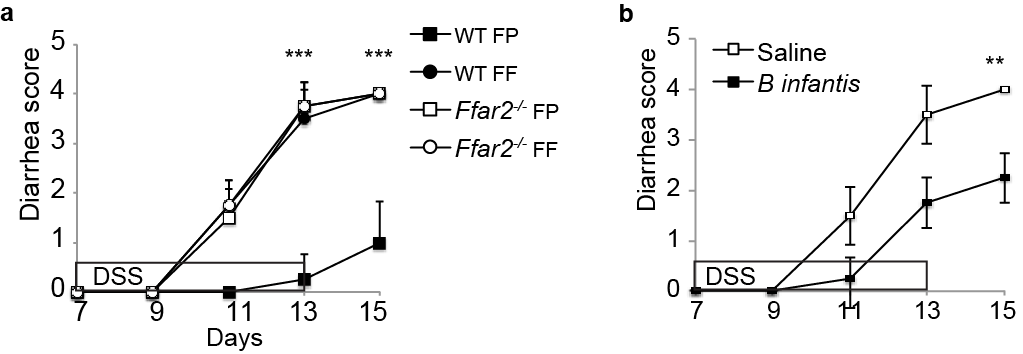


**Supplementary Figure 6.** Diarrhea in AOM/DSS treated mice in figure 6 & 7 during and after DSS challenge. *P<0.05, ** P<0.01, ** P<0.001. A representative of two experiments is shown.


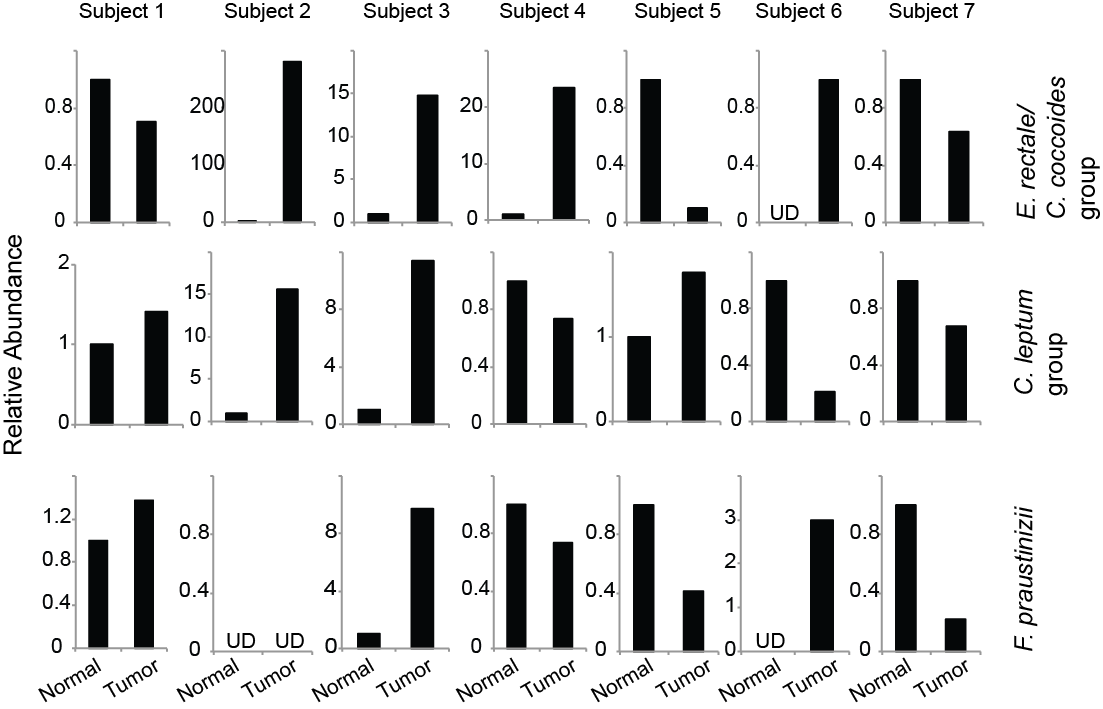


**Supplementary Figure 7.** Relative abundance of indicated bacterial groups in colon cancer tissue versus matched adjacent normal tissue is shown. UD= undetectable

**Supplementary Table 1.** Sequences of PCR primers used in this study

| Target Primer Sequences |
| --- |
| All bacteria 5’-ACTCCTACGGGAGGCAGCAGT-3’  5’-GTATTACCGCGGCTGCTGGCAC-3’  *Bacteroides* 5’-GGTTCTGAGAGGAGGTCCC-3’  5’-GCTGCCTCCCGTAGGAGT-3’  *Bifidobacterium*  5’- TCGCGTCYGGTGTGAAAG-3’  5’- CCACATCCAGCRTCCAC-3’  *E. rectale group*  5’- AAATGACGGTACCTGACTAA-3’  *(Clostridium Cluster XIVa)*  5’- CTTTGAGTTTCATTCTTGCGAA-3’  *C. leptum* group 5’- GTTGACAAAACGGAGGAAGG-3’  (*Clostridium cluster IV*) 5’- GACGGGCGGTGTGTACAA-3’  Lactobacillus group  5’-GCAGCAGTAGGGAATCTTCCA-3’  5’-GCATTYCACCGCTACACATG-3’  *H. hepaticus* 5’-GCATTTGAAACTGTTACTCTG-3’  5’-CTGTTTTCAAGCTCCCCGAAG-3’  MIB 5’-CCAGCAGCCGCGGTAATA-3’  5’**-**CGCATTCCGCATACTTCTC-3’  SFB 5’-GACGCTGAGGCATGAGAGCAT-3’  5’-GACGGCACGGATTGTTATTCA-3’  *Prevotellaceae* 5’-CCAGCCAAGTAGCGTGCA-3’  5’-TGGACCTTCCGTATTACC-3’  *TM7* 5’-GCAACTCTTTACGCCCAGT-3’  5’-GAGAGGATGATCAGCCAG-3’  *hFFAR2* 5’-GTAGCTAACACAAGTCCAGTCCT-3’  5’-CTAGGTGTTGCTTTGAAGCTTGT-3’  *mFfar2* 5’- GGCTCCCTGCCAACCTGCTG-3’  5’ GTGCACAGGGGCAGGCTGAG-3’  *Mmp-7* 5’-ACC CTG TTC TGC TTT GTG TGT C-3’  5’-TCT GAG CCT GTT CCC ACT GAT G-3’  MMP-9 5’-TTC AAG GAC GGT CGG TAT T-3’  5’-CTC TGA GCC TAG ACC CAA CTT A-3’  MMP-10 5’-TCC AGG AGT TGA GCC TAA GGT-3’  5’-CGC CTA GCA ATG TAA CCA GC-3’  IL-17A 5’-GCT CCA GAA GGC CCT CAG A-3’  5’-AGC TTT CCC TCC GCA TTG A-3’  IL-1B 5’-CCC AAC TGG TAC ATC AGC AC-3’  5’-TCT GCT CAT TCA CGA AAA GG-3’  CCL2 5’-TGC TAC TCA TTC ACC AGC AA-3’  5’-GTC TGG ACC CAT TCC TTC TT-3’  IL-12a 5’-TCT CCC ACA GGA GGT TTC TG-3’  5’-ACA GAG TTC CAG GCC ATC AA-3’  COX-2 5’-TGGCTGCAGAATTGAAAGCCCT 3'  5’-AAGGTGCTCGGCTTCCAGTATT-3' |
